# Stumbling Reactions in Partial Gravity – Neuromechanics of Compensatory Postural Responses and Inter-Limb Coordination During Perturbation of Human Stance

**DOI:** 10.3389/fphys.2019.00576

**Published:** 2019-05-21

**Authors:** Ramona Ritzmann, Kathrin Freyler, Michael Helm, Janek Holubarsch, Albert Gollhofer

**Affiliations:** ^1^Institute of Sport and Sport Science, University of Freiburg, Freiburg, Germany; ^2^Praxisklinik Rennbahn AG, Muttenz, Switzerland

**Keywords:** reflex, reduced gravity, balance, electromyography, kinematics, joint, contralateral

## Abstract

Spontaneous changes in gravity play a significant role in interplanetary space missions. To preserve the astronauts’ capability to execute mission-critical tasks and reduce the risk of injury in transit and on planetary surfaces, a comprehensive understanding of the neuromuscular control of postural responses after balance deterioration in hypo- or hyper-gravity conditions is essential. Therefore, this study aimed to evaluate the effect of acute gravitational variation on postural adjustments in response to perturbations. Gravitational changes were induced using parabolic flight. Postural set was manipulated by randomly providing unilateral left, bilateral or split perturbations which require balance corrections to restore postural stability. In six subjects, postural reactions were recorded after anterior and posterior surface perturbations for progressively increased gravitational conditions spanning from 0.25 to 1.75 g. Ankle and knee joint kinematics and electromyograms (EMG) of eight leg muscles were recorded prior (PRE) and after perturbation onset. Muscle activation onset latencies and amplitudes in the short-, medium-, and long-latency responses (SLR, MLR, LLR) were assessed. Results demonstrate an increased muscle activity (*p* < 0.05) and co-contraction in the lower extremities (*p* < 0.05) prior to perturbation in hypo- and hyper-gravity. After perturbation, reduced muscle onset latencies (*p* < 0.05) and increased muscle activations in the MLR and LLR (*p* < 0.05), concomitant with an increased co-contraction in the SLR, were manifested with a progressive rise in gravity. Ankle and knee joint deflections remained unaffected, whereas angular velocities increased (*p* < 0.05) with increasing gravitation. Effects were more pronounced in bi- compared to unilateral or split perturbations (*p* < 0.05). Neuro-mechanical adaptations to gravity were more distinct and muscle onset latencies were shorter in the displaced compared to the non-displaced leg. In conclusion, the timing and magnitude of postural reflexes involved in stabilization of bipedal stance are gravity-dependent. The approximately linear relationship between gravity and impulse-directed EMG amplitudes or muscle onset latencies after perturbation indicates that the central nervous system correctly predicts the level of gravity. Moreover, it accurately governs contractions in the antigravity musculature to counterbalance the gravitational pull and to regain upright posture after its disturbance. Importantly, unilateral perturbations evoked fast reflex responses in the synergistic muscles of the non-displaced contralateral leg suggesting a synchronized inter-limb coordination mediated by spinal circuitries.

## Introduction

The control of bipedal posture and gait and the capacity to regain equilibrium after its deterioration in variable environments is a crucial prerequisite for the success of future manned space discovery (White and Averner, 2001). Surface space walks on the neighboring planets and exploratory activities (Minetti, 2001) require a safe control of the habitual orthograde postural equilibrium with a high demand on the central nervous system (CNS) to immediately adapt muscle forces in accordance with gravity (Mergner and Rosemeier, 1998). With a range from 0 g up to 2 g, scenarios of interplanetary space travel within our solar system will expose humans to habitats where it is imperative to sustain great forces (Ritzmann et al., 2015) or deal with low friction and slippery ground surfaces (Minetti, 2001; Pavei and Minetti, 2016).

In recent debates, scientists have postulated the gravity sensitivity of bipedal stance and gait (Mergner and Rosemeier, 1998; Minetti et al., 2012a). Simulation studies exposing humans to changes in gravity used partial or additional weight-bearing (Hwang et al., 2011; Freyler et al., 2014), water buoyancy (Minetti et al., 2012a) and hypo- or hyper-gravity (Miyoshi et al., 2003). These experiments determined that spontaneous changes in gravity have a significant impact on posture and movement control associated with changes in joint torque (Mergner and Rosemeier, 1998) and neuromuscular activity (Ali and Sabbahi, 2000; Miyoshi et al., 2003). Assuming a constant mass, weight (force) is proportional with gravity (acceleration) based on the equation *F* = *m*^∗^*a*. Thereby, adaptations in somatosensory feedback (Layne et al., 2001) and compensatory reflex activation have been found (Nakazawa et al., 2004; Ritzmann et al., 2015). To date, there is scarce scientific evidence regarding neuromuscular recovery responses to sudden perturbations in such unknown gravity conditions (Ritzmann et al., 2015).

On Earth, gravity provides the reference for spatial orientation that is sensed by the otolith organs and indirectly by the somatosensory system (Nashner and Berthoz, 1978). Studies dealing with compensatory postural responses in fall situations showed that reflexive muscle activations provide appropriate joint torques for an immediate re-stabilization of the center of mass (COM) (Taube et al., 2008). This physiological model is characterized by phase-specific reflex components defined as short- (SLR), medium- (MLR), and long-latency responses (LLR) following the onset of perturbation, indicating different control levels within the CNS that govern the reflectory activation (Horak and Nashner, 1986; Jacobs and Horak, 2007). The temporal distinction of these responses, meaning their onset latency and modulation capacity on specific levels within the CNS, is of functional significance for human stance control (Taube et al., 2006). For instance, slight postural disturbances (i.e., small ankle joint rotations) are compensated by immediate, non-functional monosynaptic stretch reflex responses in the SLR (Gollhofer and Rapp, 1993). Thereby, fast length changes within the muscle elicited by the perturbation of the surface are detected by the muscle spindles and subsequently evoke a muscular response occurring 30–50 ms after perturbation onset (Honeycutt et al., 2012). However, those quick responses are mostly unfunctional, as they are mainly controlled spinally and hence occur too fast to be modulated by supraspinal areas (Dietz, 1992). Studies investigating stance perturbations could further demonstrate that functionally crucial muscle activation (>65 ms after onset, MLR and >85 ms after onset, LLR) occurs when the COM is shifted away from the vertical provoking a quite challenging postural instability (Dietz et al., 1989a, 1991; Gollhofer et al., 1989). Those reflex responses are supposed to be attributed to polysynaptic reflexes via II-afferents, and it is assumed that the CNS intervenes in the spinal pathway by integrating a central control from supraspinal levels to modulate the muscular response appropriately (Jacobs and Horak, 2007; Tokuno et al., 2009).

To control the entire body, neuromuscular activity is synchronized by inter-limb coordination in gait and stance control (Dietz and Berger, 1984; Berger et al., 1987). This is true for bilateral postural disturbances induced by mechanical perturbations, but surprisingly has also manifested for synergistic muscles of both legs when exposed to single mono-lateral perturbations (Dietz et al., 1989a; Habib Perez et al., 2016). This temporal synchronization of electromyograms from each limb in the orthograde stance emphasizes the integrity of the CNS in utilizing contralateral contribution for regulation of the COM within its base of support by producing a symmetric agonistic activation in both limbs (Dietz et al., 1989a). In addition to the ipsi- and contralateral muscle synergies, authors postulate the interplay of antagonistic muscles encompassing the joints of the lower extremity in determination of the quality and safety of postural equilibrium (Rosa, 2015). An increased antagonistic co-activation has been observed to be a major factor in reducing the range of motion (Ritzmann et al., 2015) while mechanically stabilizing joints as a safety and injury prevention mechanism (Tucker et al., 2008; Nagai et al., 2013).

Contradictory results have been observed regarding the timing (Dietz et al., 1989b; Poyhonen and Avela, 2002) and magnitude of reflex responses (Nomura et al., 2001; Nakazawa et al., 2004) Findings in kinematics and intra- and inter-limb coordination are heterogenous as well (Phadke et al., 2006; Hwang et al., 2011). Therefore, despite the substantial number of articles related to this subject, the underlying neuromuscular mechanisms in view of bi-pedal leg coordination and their functional consequences for the control of posture are poorly understood. It is emphasized that differing methodologies among the gravity-simulating studies may have caused confounding effects such as changes in friction (Dietz et al., 1989b), artificial stabilization (Freyler et al., 2014), hydrostatic pressure (Thornton et al., 1992), and inertia (Mergner and Rosemeier, 1998) leading to a reduced validity or reliability between the measurements (Dietz et al., 1992). To minimize this overlap, auspicious test conditions can be achieved in space-like environments by a gradual change of gravitational force itself in parabolic flights (Mergner and Rosemeier, 1998; Pletser et al., 2012).

The purpose of this study was to elucidate the gravity-dependence of bipedal human stance based on the physiological model of recovery responses to external perturbation. With reference to Dietz et al. (1989a), we set an emphasis on the timing and magnitude of neuromuscular responses coupled with their topographic distinction related to the functional significance of inter-limb coordination (Habib Perez et al., 2016). For that purpose, we recorded electromyograms in the upper and lower limb muscles (Taube et al., 2008), as well as kinematics of both limbs during translational surface perturbations of different modes (physics of bi-, unilateral and split perturbations) during parabolic flight including partial gravity levels. It was hypothesized that acute changes in gravity affect the postural response after perturbation and is required to anticipate neuromuscular control in timing and magnitude. We expected that a gradual increase in gravity from 0.25 to 1.75 g would result in a gradual increase in limb muscle activation intensities and faster muscle onset latencies in response to the perturbation stimulus. We further expected that a phase- and leg-specific reflex adjustment would compensate for the changes in gravitational loading. Three sub-hypotheses have been derived with reference to Dietz et al. (1989a) and Ritzmann et al. (2015): we expect (1) the MLR and LLR to be most affected by changes in gravity, (2) a distinct inter-limb synchronization of neuromuscular activation in response to perturbation and (3) an increase in antagonistic co-activation below and above 1 g.

## Materials and Methods

### Subjects

Six subjects (two females, four males, height 173 ± 6 cm, body mass 66 ± 8 kg, age 33 ± 8 years old) participated in this study. All participants gave written informed consent to the experimental procedure, which was in accordance with the latest revision of the Declaration of Helsinki and approved by the French authorities responsible for the protection of subjects participating in biomedical research (DEMEB of the AFSSAPS) as well as the ethical committee of the University of Freiburg (89/12). The participants underwent two obligatory medical investigations and were healthy with no previous neurological irregularities or injuries of the lower extremities. Exclusion criteria were pregnancy, sickness, injuries, vestibular or proprioceptive dysfunction, fear of flying, previous surgeries on the left or right leg, neurodegenerative diseases or single events associated with neural dysfunctions and an age >41 years.

### Study Design

**FIGURE 1:**
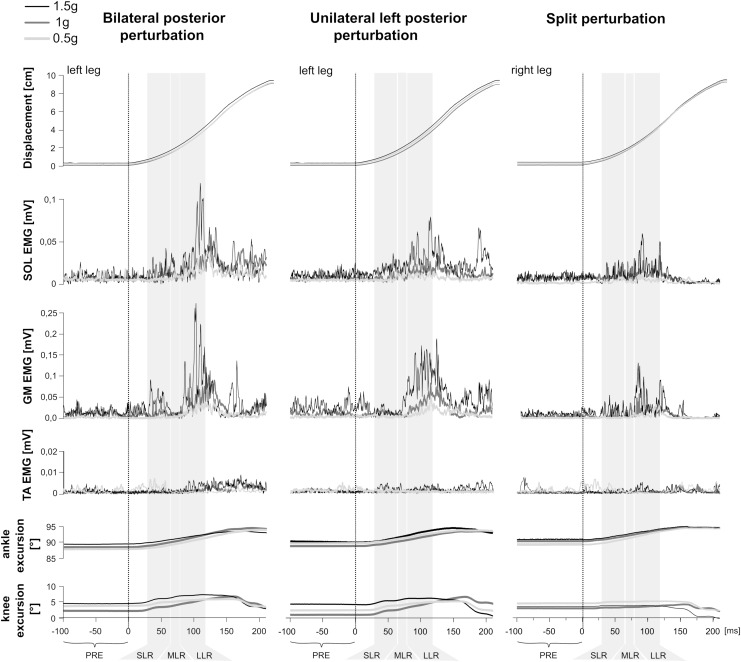
Examples of bilateral **(Left)**, unilateral left **(Middle)**, and split **(Right)** perturbations illustrated by a representative subject. At the top, the trajectory of the platform displacement is illustrated. Below illustrates modulations of the rectified and averaged electromyograms (EMG) of the shank muscles M. soleus (SOL), gastrocnemius medialis (GM) and tibialis anterior (TA) as well as angle and knee joint excursions (bottom) for three gravity conditions that denote hypo-gravity (0.5 g, light gray) Earth gravity (1 g, dark gray) and hyper-gravity (1.5 g, black). Data comprises the means from a minimum of five perturbations for each gravity level. The vertical dashed line indicates the onset of the mechanical displacement. Also marked are the relevant EMG phases: pre-activity (PRE) -100–0 ms before perturbation onset, SLR 30–60 ms, MLR 60–85 ms and LLR 85–120 ms after perturbation onset. Muscular activity progressively increased while muscle activation onset latencies shortened with increasing gravity.

A single-group repeated-measures study design was used to examine differences between postural responses to perturbations in Earth gravity (1 g) with those delivered in hypo- (0.25 g, 0.5 g, and 0.75 g) and hyper-gravity (1.25 g, 1.5 g, and 1.75 g) on the basis of leg joint kinematics and electromyograms (EMG) of lower limb muscles (Figure 1). Measurements were performed barefoot on a two-belt treadmill which generated either bi-lateral or unilateral left or split perturbations separated by 3–5 s breaks. Prior to perturbations, subjects stood upright with knee and hip joint extended, arms hanging at the lateral sides and weight equally distributed over both feet.

The order of the recordings in 1 g, hypo- and hyper-gravity was pseudo-randomized between subjects to control for confounding effects such as habituation. Fatigue was avoided by rest pauses (∼2 min) in between the parabolas.

Before the measurements, subjects performed three isometric maximal voluntary contractions (MVCs) for each recorded muscle, according to Wiley and Damiano (1998) and Roelants et al. (2006); the trial with the highest EMG was used for data normalization. The MVCs were executed against resistance for 3 s with recovery pauses of 1 min between trials and repetitions. Body position during MVCs was strictly controlled and supervised through goniometric recordings with standardized knee and hip joint angles by the authors. Antagonistic muscle activation was monitored, and trials were repeated when antagonists were activated.

### Parabolic Flights

**FIGURE 2:**
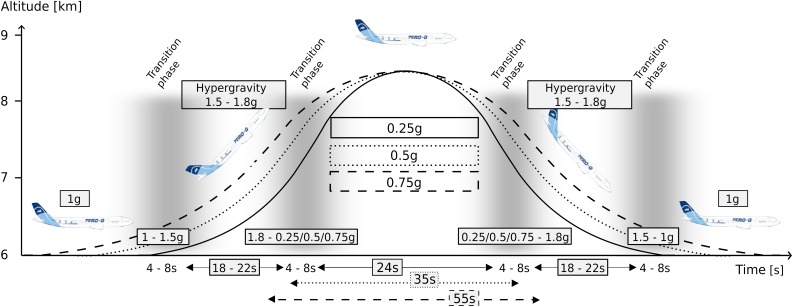
The parabolic flight maneuver and corresponding gravity levels. The level flight (1 g) becomes a steep climb flight inducing hyper-gravity (1.1–1.8 g), followed by hypo-gravity (0.25, 0.5, or 0.75 g) and another hyper-gravity phase before returning to a level flight (1 g), also comprising the respective transition phases between 1 and 1.8 g as well as between the respective hypo-gravity levels and 1.8 g. The maneuver was repeated 31 times in a random order for each of the three flight days. The different gravitational conditions were clustered into equidistant intervals and neuromuscular control and joint kinematics were assessed after surface perturbations.

Gravitational transition was induced using parabolic flight. The experiments were conducted aboard the ZERO-G aircraft (Novespace, Bordeaux, France) during the 1st International Space Life Sciences Working Group Parabolic Flight Campaign (IPFC). This campaign comprised three flight days; each flight lasted 3 h and comprised 31 parabolas for experimentation. Per flight two subjects were measured for 15 parabolas each. The course of one parabola is illustrated in Figure 2. The partial gravity periods were embedded within two hyper-gravity (1–1.8 g) periods lasting approximately 15–20 s, wherein 10 parabolas included 24-s of 0.25 g, 10 parabolas included 35-s of 0.5 g and 10 parabolas included 55-s of 0.75 g.

Before each flight day, two participants were prepared for the measurements and were given a sex-weight-based injection of 0.2–0.7 ml of Scopolamine 30 min before takeoff to prevent motion sickness (Ritzmann et al., 2016).

### Perturbations

**FIGURE 3:**
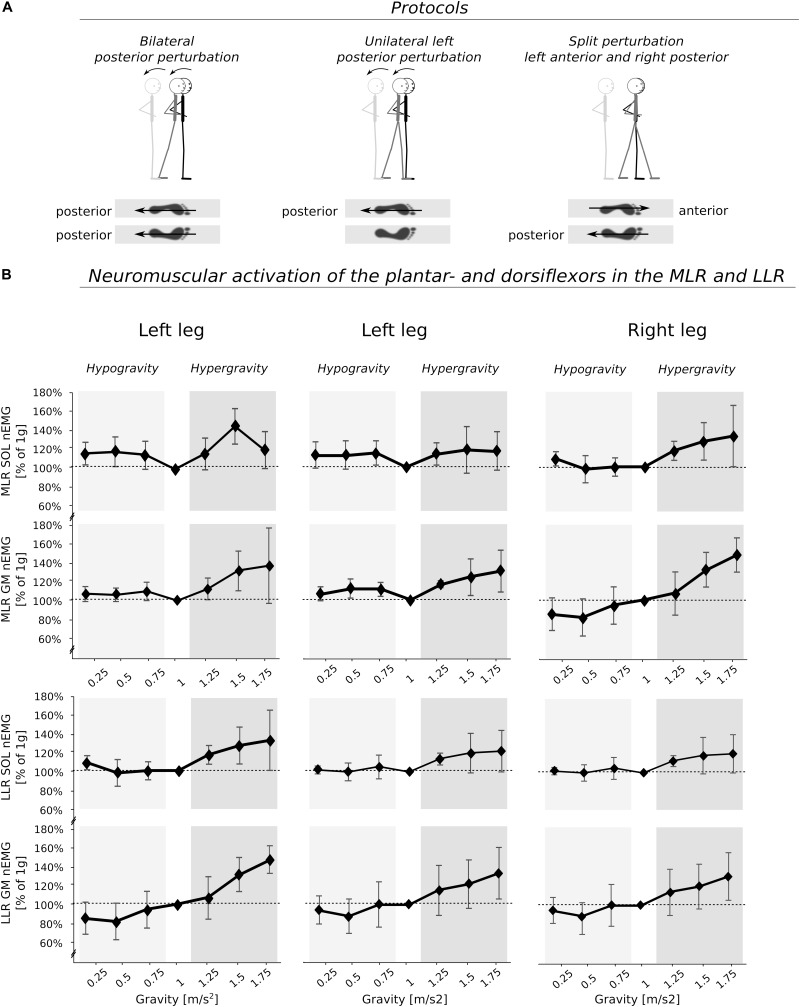
**(A)** Depiction of the perturbation protocols which were used for analysis as well as **(B)** the means of the electromyograms (EMG) during the medium- and long-latency responses (MLR and LLR) for the plantarflexors [M. soleus (SOL) and M. gastrocnemius medialis (GM)] of the left leg and right leg displayed for the seven gravity levels that span equidistant from hypo- to Earth to hyper-gravity. The MVC normalized iEMG (nEMG) is further normalized to the reference values obtained during the measurements in 1 g. Independent of the postural set (bi-, unilateral left or split perturbation), neuromuscular activity increased progressively with increasing gravity. Note that particularly high gravity levels (>1.5 g) may have led to an inhibition of the neuromuscular activity as shown by the decline in EMG for SOL. The *p*-value refers to the Friedman Test; $\eta_{p}^{2}$ displays the effect sizes.

**FIGURE 4:**
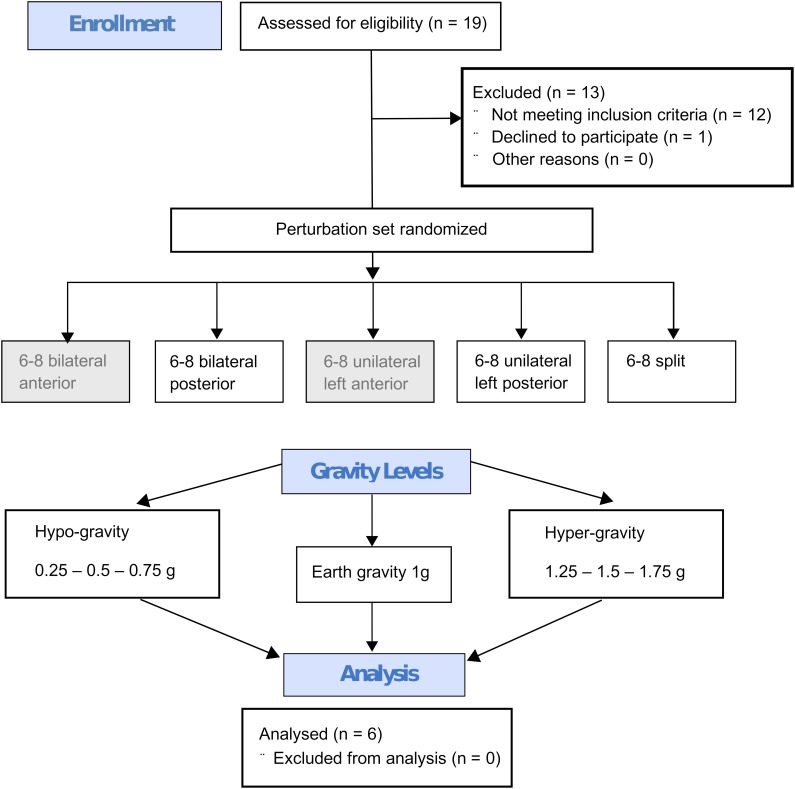
Flow diagram describing the study design with the steps from enrollment to the randomized perturbation modes applied in seven different gravity levels to the analysis. Every subject performed six to eight repetitions under identical conditions (perturbation mode and g-level) and the mean value of these repetitions per subject and g-level were calculated. This mean value was then used for the Friedman test; the statistics for the respective g-levels were calculated separately for all recorded muscles of the left and right leg, phases and perturbation modes with *n* = 6 subjects. The anterior perturbations (gray background) were only applied for randomization purposes and were not included in the analysis.

Sudden and unexpected acceleration impulses were applied independently to the two belts of the treadmill (physical parameters in Table 1). Five different modes of perturbations were elicited in a random order according to Dietz et al. (1989a): (1) simultaneous bilateral anterior perturbation, (2) simultaneous bilateral posterior perturbation, (3) unilateral anterior perturbation on the left leg only, (4) unilateral posterior perturbation on the left leg only, and (5) simultaneous bilateral perturbation in opposing directions. For data analysis, perturbations 2, 4, and 5 were used (Figure 3A). The anterior perturbations (1, 3) were applied for the purpose of randomization in order to minimize preparation possibilities for the subjects. As they had to prepare for balance disturbances coming from either anterior or posterior, the initial stance position had to be neutral. The five perturbation modes were applied in random order during seven gravity levels (0.25 g, 0.5 g, 0.75 g, 1 g, 1.25 g, 1.5 g, and 1.75 g). The randomization sequence of the perturbations was performed by software (Labview, Imago, Pfitec, Freiburg). Per g-level, we recorded six to eight perturbations for each of the three analyzed perturbation modes for each subject (Figure 4). Hence, we in total recorded approximately 280 perturbations per subject. The mechanical displacement was assessed by a potentiometer (sampling frequency 1 kHz). Subjects wore a safety harness for fall avoidance which was attached to the aircraft ceiling.

**Table 1:** Physical parameters of treadmill perturbations.

	Bilateral anterior		Bilateral posterior		Unilateral left anterior		Unilateral left posterior		Split	
					
	Left leg	Right leg	Left leg	Right leg	Left leg	Right leg	Left leg	Right leg	Left leg	Right leg
*d* (cm)	10.0 ± 0.0^≈^	10.0 ± 0.0^≈^	-10.0 ± 0.0^≈^	-10.0 ± 0.0^≈^	10.0 ± 0.0^≈^	-	-10.0 ± 0.0^≈^	-	-10.0 ± 0.0^≈^	10.0 ± 0.0^≈^
*t* (ms)	212 ± 3^≈^	211 ± 4^≈^	214 ± 5^≈^	210 ± 2^≈^	209 ± 5^≈^	-	214 ± 4^≈^	-	211 ± 2^≈^	210 ± 4^≈^
*v*_max_ (m/s)	0.8 ± 0.0^≈^	0.8 ± 0.0^≈^	0.8 ± 0.0^≈^	0.8 ± 0.0^≈^	0.8 ± 0.0^≈^	-	0.8 ± 0.0^≈^	-	0.8 ± 0.0^≈^	0.8 ± 0.0^≈^
*a*_max_ (m/s^2^)	21 ± 1^≈^	22 ± 0^≈^	22 ± 3^≈^	21 ± 1^≈^	21 ± 1^≈^	-	22 ± 3^≈^	-	22 ± 3^≈^	23 ± 1


*Values are absolute values. [*v*_*max*_, maximum speed (m/s); *t*, impulse duration (ms); *a*_*max*_, maximal acceleration of the treadmill (m/s^*2*^); *d*, distance (cm)].*

### Selection of Gravity Levels and Trial Criteria

The g-level was monitored by an accelerometer (sampling frequency 1 kHz). For each subject, we recorded five parabolas per hypo-gravity level (0.25 g, 0.5 g, and 0.75 g). Further, for each of the five parabolas, the two hyper-gravity phases as well as the transition phases (Figure 2) were used to record the postural reactions in the hyper-gravity levels (1.25 g, 1.5 g, and 1.75 g). Measurements during steady flight served as a reference in 1 g.

We applied two standardized selection criteria to consider the trial as a valid one without postural interference beyond the mechanical surface translation itself due to pilot, weather or aircraft factors: First, the g-data were extracted for the predefined g-level and needed to lay within boundaries of ±0.1 g for a time interval of 350 ms (from 100 ms prior to until 250 ms after perturbation onset, Dietz et al., 1989a). If the flight maneuver was instable due to turbulences on the vertical plane or the boundaries of 0.1 g were exceeded, the perturbation trial was excluded. Second, the initial stance position of the subjects prior to each perturbation determined by the COM position was controlled by a camera [sampling frequency 60 Hz (GoPro, HERO 3, San Mateo, CA, United States)]. If the COM trajectory in the horizontal plane was not within the acceptable bounds of the 90% confidence interval (CI) from trials assessed in 1 g obtained as reference values, the trial was excluded as the initial starting position was considered to be unstable throughout changes in aircraft thrust. In total, we had to exclude nine trials within all six subjects based on one of the aforementioned criteria.

### Outcome Measures

#### Electromyography (EMG)

Bipolar Ag/AgCl surface electrodes (Ambu Blue Sensor P, Ballerup, Denmark, diameter 9 mm, center-to-center distance 34 mm) were placed over the musculus soleus (SOL l), gastrocnemius medialis (GM l), tibialis anterior (TA l), vastus medialis (VM l) and biceps femoris (BF l) of the left leg and over the musculus soleus (SOL r), gastrocnemius medialis (GM r) and tibialis anterior (TA r) of the contralateral right leg. The longitudinal axes of the electrodes were in line with the presumed direction of the underlying muscle fibers. The reference electrode was placed on the patella. Interelectrode resistance was kept below 2 kΩ by means of shaving, light abrasion and degreasing of the skin with a disinfectant. Procedures were executed according to SENIAM (Hermens et al., 2000). The EMG signals were transmitted via shielded cables to the amplifier (band-pass filter 10 Hz to 2 kHz, 200× amplified) and recorded with 1 kHz (A/D-conversion via a National Instruments PCI-6229 DAQ-card, 16 bit resolution).

#### Kinematics

Ankle (dorsiflexion and plantarflexion) and knee (flexion and extension) joint kinematics of the left as well as the contralateral leg in the sagittal plane were recorded by electro-goniometers (Biometrics, Gwent, United Kingdom). The ankle goniometers were fixed at the lateral aspect of the ankle, with its movable endplates attached parallel to the major axis of the foot, in line with the fifth metatarsal, and the major axis of the leg, in line with the fibula. The knee goniometers were placed over the lateral epicondyle of the femur, with one endplate attached to the shank, aligned to the lateral malleolus of the fibula and the other to the thigh, aligned to the greater trochanter. The knee angle was set to zero at 0° during normal upright stance, and joint flexion was reflected by an angle >0°. An angle of 90° between the fifth metatarsal and the fibula corresponded to a 90° ankle angle; an angle >90° reflected plantarflexion. Signals were recorded with a sampling frequency of 2 kHz.

To control the starting position before each perturbation, we monitored the vertical COM projection with 2D video recordings at a distance of 3 m from the treadmill perpendicularly to the sagittal plane (Gambelli et al., 2016). The vertical COM projection was determined using the software SIMI Motion (Unterschleißheim, Germany). Markers were taped on the participants’ skin on the anatomical landmark iliac crest representative for the COM.

### Data Processing

The joint angles were determined at the onset of perturbation, and the angular joint excursions (°) were calculated from onset of perturbation until 250 ms after the onset. The angular velocity of joint excursions was assessed as follows: v_joint_ = *x*^∗^*t* with *x* describing the displacement [°] and *t* the time to maximal excursion [s] in a timeframe of 0–210 ms.

EMG signals were rectified, averaged, integrated (iEMG) and time-normalized for four time intervals, based on previously reported onset latencies and durations of the reflex components (Lee and Tatton, 1975; Marsden et al., 1978; Sinkjaer et al., 1999): the pre-activation phase (PRE, -100–0 ms before perturbation), the SLR (30–60 ms after perturbation), MLR (60–85 ms after perturbation) and LLR (85–120 ms after perturbation) (Hobara et al., 2008; Taube et al., 2008; Zuur et al., 2010). These integrals were normalized to the MVC of the corresponding muscle. Furthermore, antagonistic co-activation of TA and SOL (TA_SOL), TA and GM (TA_GM) and VM and BF (VM_BF) were calculated for the pre-activation phase for both legs (Hoffrén et al., 2011). Furthermore, muscle activation onset latencies of each muscle after the perturbation were identified as the first burst >2 standard deviations above the baseline EMG and displayed in ms (Henry et al., 1998).

All data were averaged for identical perturbation modes.

### Statistics

Data are presented as means ± standard deviations (SD) for each g-level. We used a two-step procedure (Figure 4): first, as every subject performed six to eight repetitions under identical conditions (perturbation mode and g-level), we calculated the mean value of these repetitions per subject and g-level for data reduction. Second, this synthesized mean value per subject and g-level was used for the Friedman test. The statistics for the respective g-levels were calculated separately for all recorded muscles of the left and right leg, phases and perturbation modes with *n* = 6 subjects. Standard errors (SEs) were calculated across gravity levels.

To evaluate kinematic and neuromuscular modulations in response to changes in gravitational loading [gravity (7)], the Friedman Test was used for *n* = 6 subjects. The dependent variables were the onset latencies and EMGs normalized to MVC for PRE, SLR, MLR, and LLR for the eight recorded muscles of the lateral and contralateral leg as well as ankle and knee joint excursions and velocities. The level of significance was set to *P* = 0.05. The false discovery rate was controlled according to the Benjamini-Hochberg-Yekutieli method, a less conservative but still stringent statistical approach conceptualizing the rate of type I errors (Benjamini and Hochberg, 1995; Benjamini and Yekutieli, 2005). Partial eta squared ($\eta_{p}^{2}$) was also used as an estimate of the effect size [$\eta_{p}^{2}$ < 0.01 small, 0.01 ≤$\eta_{p}^{2}$ ≤ 0.06 medium, 0.24 < $\eta_{p}^{2}$ large effect size (Cohen, 1973)].

Bivariate, two-tailed Pearson correlation analyses were conducted to determine the strength of linear relations between the two variables joint velocities and the neuromuscular activity in the reflex phases MLR and LLR, for the muscles SOL and GM, respectively. Furthermore, to compare correlations from dependent samples obtained in the seven progressively increasing gravity levels, correlation coefficients (r0.25, r0.5, r0.75, r1, r1.25, r1.5, and r1.75) were converted into a *z*-score using Fisher’s r-to-z transformation (Steiger, 1980). Subsequently, the asymptotic covariance of the estimates was computed according to Steiger (1980).

Equivalence statistics were used to determine if the physics of the treadmill perturbations were statistically equal between the gravity levels below and above 1 g compared to 1 g. For this purpose, the 95% CI was calculated for the differences between 1 g and respective gravity level. If the CI lay within the acceptable boundaries (which were determined based on the variance within the 1 g data set (Borman et al., 2009) the differences were statistically equal and the respective parameter was marked with a ≈ symbol.

All statistical analyses were conducted using SPSS 24.0 software (SPSS, Chicago, IL, United States) and calculators from Lee and Preacher (2013).

## Results

In all gravity levels and for all perturbation modes, subjects succeeded to regain their postural equilibrium after the mechanical perturbation. There was no trial, in which subjects needed to be secured or were falling.

### Physics of Treadmill Perturbation

The treadmill displacement, its maximum speed, the acceleration of the treadmill and the impulse duration for the bilateral, unilateral, and split perturbations are displayed in Table 1. The physics were statistically equal between the three perturbation conditions over the seven progressively increased gravity levels.

### Neuromuscular Activity

Means of the EMG amplitudes in the recorded shank and thigh muscles are displayed in Tables 2–4. Mean rectified EMG signal traces of a representative subject are given in Figure 1. Results demonstrate an increased muscle activity in the lower extremities (*p* < 0.05) prior to perturbation in hypo- and hyper-gravity.

**Table 2:** Bilateral posterior perturbation for seven gradually and equidistantly increasing g-levels.

	Hypo gravity			Earth gravity	Hyper gravity			Statistics		
				
	0.25 g	0.5 g	0.75 g	1 g	1.25 g	1.5 g	1.75 g	*SE*	*P*	$\eta_{p}^{2}$
PRE SOL l	1.15 ± 0.12	**1.17 ± 0.15**	**1.13 ± 0.14**	**1**	**1.17 ± 0.15**	**1.17 ± 0.11**	**1.16 ± 0.17**	**2%**	**<0.01**	**0.39**
PRE GM l	**1.12 ± 0.10**	**1.10 ± 0.12**	**1.12 ± 0.13**	**1**	**1.13 ± 0.15**	**1.15 ± 0.16**	**1.16 ± 0.14**	**3%**	**0.03**	**0.25**
PRE TA l	1.20 ± 0.18	1.16 ± 0.18	1.16 ± 0.18	1	1.16 ± 0.18	1.17 ± 0.16	1.16 ± 0.18	2%	0.12	0.07
PRE VM l	1.10 ± 0.09	1.08 ± 0.10	1.09 ± 0.10	1	1.09 ± 0.10	1.11 ± 0.09	1.08 ± 0.10	4%	0.35	0.02
PRE BF l	1.14 ± 0.14	1.13 ± 0.16	1.15 ± 0.14	1	1.15 ± 0.17	1.16 ± 0.14	1.11 ± 0.15	3%	0.19	0.05
PRE SOL r	**1.14 ± 0.14**	**1.13 ± 0.14**	**1.13 ± 0.15**	**1**	**1.14 ± 0.16**	**1.16 ± 0.14**	**1.13 ± 0.15**	**5%**	**0.01**	**0.48**
PRE GM r	1.12 ± 011	1.11 ± 0.12	1.12 ± 0.13	1	1.12 ± 0.13	1.14 ± 0.11	1.12 ± 0.12	2%	0.07	0.06
PRE TA r	1.11 ± 0.10	1.10 ± 0.11	1.10 ± 0.11	1	1.10 ± 0.11	1.13 ± 0.11	1.12 ± 0.13	3%	0.19	0.03
SLR SOL l	**1.15 ± 0.11**	**1.17 ± 0.14**	**1.13 ± 0.13**	**1**	**1.15 ± 0.15**	**1.41 ± 0.17**	**1.18 ± 0.18**	**3%**	**<0.01**	**0.56**
SLR GM l	**1.12 ± 0.09**	**1.10 ± 0.11**	**1.11 ± 0.11**	**1**	**1.13 ± 0.11**	**1.40 ± 0.19**	**1.17 ± 0.20**	**2%**	**0.02**	**0.30**
SLR TA l	1.17 ± 0.15	1.17 ± 0.19	1.15 ± 0.18	1	1.15 ± 0.18	1.36 ± 0.22	1.17 ± 0.20	4%	0.18	0.04
SLR VM l	**1.09 ± 0.08**	**1.08 ± 0.10**	**1.10 ± 0.10**	**1**	**1.09 ± 0.09**	**1.17 ± 0.13**	**1.23 ± 0.11**	**5%**	**<0.01**	**0.34**
SLR BF l	1.13 ± 0.14	1.12 ± 0.16	1.15 ± 0.14	1	1.13 ± 014	1.34 ± 0.16	1.12 ± 0.17	5%	0.12	0.09
SLR SOL r	**1.10 ± 0.13**	**1.10 ± 0.12**	**1.13 ± 0.12**	**1**	**1.13 ± 0.15**	**1.42 ± 0.16**	**1.14 ± 0.12**	**7%**	**0.01**	**0.28**
SLR GM r	**1.12 ± 0.11**	**1.11 ± 0.12**	**1.13 ± 0.14**	**1**	**1.13 ± 0.11**	**1.54 ± 0.18**	**1.13 ± 0.17**	**2%**	**0.03**	**0.17**
SLR TA r	1.10 ± 0.09	1.10 ± 0.12	1.10 ± 0.11	1	1.10 ± 0.11	1.31 ± 0.20	1.15 ± 0.16	6%	0.37	0.02
MLR SOL l	**0.99 ± 0.12**	**1.00 ± 0.08**	**1.04 ± 0.08**	**1**	**1.18 ± 0.13**	**1.20 ± 0.10**	**1.19 ± 0.25**	**4%**	**<0.01**	**0.40**
MLR GM l	**1.08 ± 0.09**	**1.07 ± 0.08**	**1.11 ± 0.11**	**1**	**1.14 ± 0.13**	**1.35 ± 0.24**	**1.42 ± 0.45**	**3%**	**<0.01**	**0.71**
MLR TA l	1.19 ± 0.19	1.15 ± 0.17	1.16 ± 0.19	1	1.17 ± 0.20	1.19 ± 0.20	1.19 ± 0.26	5%	0.21	0.03
MLR VM l	1.10 ± 0.10	1.10 ± 0.09	1.09 ± 0.10	1	1.10 ± 0.11	1.12 ± 0.12	1.08 ± 0.09	6%	0.49	0.02
MLR BF l	**1.15 ± 0.15**	**1.14 ± 0.09**	**1.16 ± 0.14**	**1**	**1.18 ± 0.14**	**1.17 ± 0.17**	**1.14 ± 0.14**	**4%**	**0.02**	**0.18**
MLR SOL r	**1.16 ± 0.18**	**1.13 ± 0.16**	**1.12 ± 0.15**	**1**	**1.19 ± 0.29**	**1.28 ± 0.30**	**1.18 ± 0.17**	**3%**	**0.03**	**0.24**
MLR GM r	**1.11 ± 0.15**	**1.09 ± 0.12**	**1.11 ± 0.17**	**1**	**1.23 ± 0.08**	**1.47 ± 0.41**	**1.38 ± 0.27**	**2%**	**<0.01**	**0.52**
MLR TA r	1.13 ± 0.12	1.13 ± 0.07	1.11 ± 0.13	1	1.12 ± 0.13	1.09 ± 0.10	1.05 ± 0.10	6%	0.42	0.02
LLR SOL l	**1.11 ± 0.11**	**1.13 ± 0.14**	**1.11 ± 0.15**	**1**	**1.10 ± 0.16**	**1.17 ± 0.14**	**1.19 ± 0.25**	**6%**	**0.01**	**0.30**
LLR GM l	**1.02 ± 0.08**	**1.01 ± 0.08**	**1.04 ± 0.08**	**1**	**1.13 ± 0.12**	**1.17 ± 0.14**	**1.19 ± 0.16**	**5%**	**0.03**	**0.17**
LLR TA l	**1.18 ± 0.16**	**1.16 ± 0.17**	**1.16 ± 0.20**	**1**	**1.16 ± 0.19**	**1.20 ± 0.18**	**1.22 ± 0.23**	**3%**	**<0.01**	**0.29**
LLR VM l	1.09 ± 0.10	1.09 ± 0.09	1.09 ± 0.10	1	1.08 ± 0.10	1.11 ± 0.10	1.09 ± 0.10	7%	0.35	0.02
LLR BF l	1.07 ± 0.12	1.10 ± 0.16	1.10 ± 0.15	1	1.15 ± 0.19	1.23 ± 0.26	1.19 ± 0.27	4%	0.28	0.04
LLR SOL r	1.06 ± 0.14	1.04 ± 0.08	1.06 ± 0.12	1	1.11 ± 0.15	1.11 ± 0.11	1.15 ± 0.17	8%	0.09	0.13
LLR GM r	**0.97 ± 0.09**	**0.98 ± 0.05**	**0.99 ± 0.07**	**1**	**1.06 ± 0.04**	**1.17 ± 0.15**	**1.16 ± 0.14**	**3%**	**<0.01**	**0.37**
LLR TA r	**1.10 ± 0.10**	**1.09 ± 0.10**	**1.11 ± 0.12**	**1**	**1.09 ± 0.10**	**1.10 ± 0.07**	**1.14 ± 0.16**	**5%**	**0.02**	**0.14**


*EMG amplitudes of all subjects (mean ± SD, *n* = 6 subjects) for the five recorded muscles of the left leg and three recorded muscles of the right leg during four phases (PRE 100 ms before treadmill perturbation until perturbation onset, SLR 30–60 ms, MLR 60–85 ms, LLR 85–120 ms after perturbation). Values are normalized to the iEMG during MVC. The values for hypo- and hyper-gravity are expressed in percent of the 1 g condition. For every leg, muscle and phase the Friedman test was applied to test the effect of the g-level on the EMG amplitude. The *p*-value refers to the Friedman Test (significant g-effects are labeled with bold letters) and $\eta_{p}^{2}$ to the effect sizes. Standard errors (SEs) over all seven gravity levels are illustrated.*

**Table 3:** Unilateral left posterior perturbation for seven gradually and equidistantly increasing g-levels.

	Hypo gravity			Earth gravity	Hyper gravity			Statistics		
				
	0.25 g	0.5 g	0.75 g	1 g	1.25 g	1.5 g	1.75 g	*SE*	*P*	$\eta_{p}^{2}$
PRE SOL l	**1.18 ± 0.16**	1.13 ± 0.13	**1.14 ± 0.14**	**1**	**1.12 ± 0.10**	**1.12 ± 0.11**	**1.15 ± 0.18**	**4%**	**0.04**	**0.14**
PRE GM l	1.11 ± 0.10	1.10 ± 0.13	1.16 ± 0.12	1	1.09 ± 0.12	1.12 ± 0.14	1.14 ± 0.18	4%	0.19	0.08
PRE TA l	1.18 ± 0.17	1.16 ± 0.19	1.09 ± 0.18	1	1.11 ± 0.09	1.14 ± 0.17	1.16 ± 0.21	3%	0.05	0.10
PRE VM l	1.09 ± 0.09	1.08 ± 0.10	1.09 ± 0.10	1	1.07 ± 0.09	1.08 ± 0.10	1.08 ± 0.10	4%	0.27	0.03
PRE BF l	1.13 ± 0.14	1.12 ± 0.16	1.14 ± 0.15	1	1.11 ± 0.16	1.11 ± 0.15	1.11 ± 0.15	6%	0.41	0.02
PRE SOL r	1.14 ± 0.14	1.12 ± 0.15	1.13 ± 0.14	1	1.10 ± 0.14	1.13 ± 0.15	1.12 ± 0.16	2%	0.15	0.05
PRE GM r	1.12 ± 0.11	1.10 ± 0.13	1.12 ± 0.13	1	1.09 ± 0.12	1.12 ± 0.12	1.12 ± 0.14	7%	0.45	0.02
PRE TA r	1.11 ± 0.10	1.09 ± 0.11	1.11 ± 0.11	1	1.09 ± 0.12	1.10 ± 0.11	1.12 ± 0.14	4%	0.35	0.04
SLR SOL l	**1.12 ± 0.14**	**1.12 ± 0.12**	**1.14 ± 0.15**	**1**	**1.13 ± 0.09**	**1.17 ± 0.21**	**1.16 ± 0.16**	**2%**	**0.02**	**0.24**
SLR GM l	**1.12 ± 0.12**	**1.11 ± 0.12**	**1.13 ± 0.13**	**1**	**1.10 ± 0.08**	**1.25 ± 0.28**	**1.25 ± 0.23**	**3%**	**<0.01**	**0.36**
SLR TA l	1.17 ± 0.16	1.14 ± 0.18	1.16 ± 0.18	1	1.12 ± 0.16	1.23 ± 0.21	1.14 ± 0.18	5%	0.05	0.13
SLR VM l	1.10 ± 0.10	1.09 ± 0.10	1.09 ± 0.10	1	1.07 ± 0.09	1.18 ± 0.18	1.09 ± 0.12	4%	0.37	0.05
SLR BF l	1.13 ± 0.14	1.12 ± 0.17	1.15 ± 0.16	1	1.12 ± 0.18	1.20 ± 0.21	1.10 ± 0.14	4%	0.63	0.01
SLR SOL r	1.15 ± 0.14	1.13 ± 0.14	1.14 ± 0.14	1	1.09 ± 0.15	1.24 ± 0.19	1.13 ± 0.15	6%	0.05	0.11
SLR GM r	**1.11 ± 0.11**	**1.09 ± 0.13**	**1.11 ± 0.13**	**1**	**1.07 ± 0.13**	**1.25 ± 0.23**	**1.15 ± 0.16**	**3%**	**0.02**	**0.19**
SLR TA r	1.10 ± 0.11	1.10 ± 0.12	1.11 ± 0.11	1	1.10 ± 0.14	1.17 ± 0.19	1.10 ± 0.12	4%	0.40	0.02
MLR SOL l	**1.14 ± 0.13**	**1.11 ± 0.14**	**1.10 ± 0.12**	**1**	**1.06 ± 0.11**	**1.13 ± 0.23**	**1.12 ± 0.19**	**6%**	**0.03**	**0.21**
MLR GM l	**1.07 ± 0.08**	**1.13 ± 0.11**	**1.12 ± 0.08**	**1**	**1.18 ± 0.04**	**1.26 ± 0.21**	**1.33 ± 0.24**	**6%**	**<0.01**	**0.52**
MLR TA l	1.16 ± 0.17	1.18 ± 0.20	1.16 ± 0.17	1	1.13 ± 0.18	1.20 ± 0.20	1.16 ± 0.21	4%	0.36	0.04
MLR VM l	1.09 ± 0.08	1.10 ± 0.14	1.09 ± 0.10	1	1.08 ± 0.10	1.12 ± 0.12	1.08 ± 0.09	7%	0.61	0.01
MLR BF l	1.13 ± 0.14	1.11 ± 0.17	1.14 ± 0.14	1	1.12 ± 0.15	1.17 ± 0.16	1.10 ± 0.14	3%	0.10	0.07
MLR SOL r	**1.14 ± 0.14**	**1.13 ± 0.16**	**1.14 ± 0.15**	**1**	**1.11 ± 0.17**	**1.16 ± 0.19**	**1.13 ± 0.13**	**4%**	**0.04**	**0.09**
MLR GM r	**1.12 ± 0.11**	**1.12 ± 0.14**	**1.12 ± 0.11**	**1**	**1.08 ± 0.20**	**1.21 ± 0.21**	**1.20 ± 0.17**	**3%**	**0.03**	**0.09**
MLR TA r	1.14 ± 0.13	1.16 ± 0.12	1.11 ± 0.11	1	1.14 ± 0.21	1.15 ± 0.24	1.15 ± 0.21	3%	0.08	0.05
LLR SOL l	**1.02 ± 0.04**	**1.00 ± 0.09**	**1.05 ± 0.12**	**1**	**1.13 ± 0.06**	**1.19 ± 0.20**	**1.21 ± 0.21**	**8%**	**<0.05**	**0.63**
LLR GM l	**0.93 ± 0.18**	**0.85 ± 0.22**	**1.00 ± 0.29**	**1**	**1.18 ± 0.32**	**1.26 ± 0.31**	**1.40 ± 0.33**	**4%**	**<0.01**	**0.42**
LLR TA l	1.18 ± 0.16	1.04 ± 0.14	1.16 ± 0.18	1	1.11 ± 0.15	1.10 ± 0.21	1.16 ± 0.17	3%	0.28	0.03
LLR VM l	1.10 ± 0.10	1.00 ± 0.10	1.05 ± 0.08	1	1.06 ± 0.09	1.05 ± 0.16	1.08 ± 0.10	5%	0.47	0.01
LLR BF l	1.13 ± 0.14	1.11 ± 0.13	1.15 ± 0.16	1	1.08 ± 0.17	1.09 ± 0.16	1.10 ± 0.15	2%	0.31	0.02
LLR SOL r	1.07 ± 0.13	1.03 ± 0.13	0.98 ± 0.12	1	1.12 ± 0.14	1.11 ± 0.17	1.16 ± 0.15	7%	0.05	0.06
LLR GM r	**1.04 ± 0.12**	**1.00 ± 0.14**	**0.98 ± 0.14**	**1**	**1.15 ± 0.09**	**1.19 ± 0.25**	**1.28 ± 0.14**	**6%**	**<0.01**	**0.42**
LLR TA r	1.10 ± 0.10	1.00 ± 0.13	1.11 ± 0.12	1	1.06 ± 0.08	1.07 ± 0.14	1.10 ± 0.11	4%	0.49	0.02


*EMG amplitudes of all subjects (mean ± SD, *n* = 6 subjects) for the five recorded muscles of the left leg and three recorded muscles of the right leg during four phases (PRE 100 ms before treadmill perturbation until perturbation onset, SLR 30–60 ms, MLR 60–85 ms, LLR 85–120 ms after perturbation). Values are normalized to the iEMG during MVC. The values for hypo- and hyper-gravity are expressed in percent of the 1 g condition. For every leg, muscle and phase the Friedman test was applied to test the effect of the g-level on the EMG amplitude. The *p*-value refers to the Friedman Test (significant g-effects were labeled with bold letters) and $\eta_{p}^{2}$ to the effect sizes. Standard errors (SEs) over all seven gravity levels are illustrated.*

**Table 4:** Split perturbation (left leg anterior and right leg posterior) for 7 gradually and equidistantly increasing g-levels.

	Hypo gravity			Earth gravity	Hyper gravity			Statistics		
				
	0.25 g	0.5 g	0.75 g	1 g	1.25 g	1.5 g	1.75 g	*SE*	*P*	$\eta_{p}^{2}$
PRE SOL l	1.16 ± 0.13	**1.17 ± 0.17**	**1.14 ± 0.16**	**1**	**1.14 ± 0.15**	**1.09 ± 0.10**	**1.18 ± 0.25**	**2%**	**0.02**	**0.28**
PRE GM l	1.12 ± 0.11	1.09 ± 0.11	1.12 ± 0.13	1	1.15 ± 0.23	1.13 ± 0.15	1.15 ± 0.21	1%	0.07	0.12
PRE TA l	**1.20 ± 0.18**	**1.14 ± 0.17**	**1.17 ± 0.19**	**1**	**1.13 ± 0.17**	**1.15 ± 0.20**	**1.16 ± 0.18**	**3%**	**0.01**	**0.24**
PRE VM l	1.09 ± 0.09	1.07 ± 0.09	1.09 ± 0.10	1	1.07 ± 0.09	1.08 ± 1.10	1.08 ± 0.10	2%	0.38	0.04
PRE BF l	**1.14 ± 0.14**	**1.11 ± 0.14**	**1.15 ± 0.15**	**1**	**1.10 ± 0.15**	**1.11 ± 0.14**	**1.11 ± 0.15**	**4%**	**0.02**	**0.19**
PRE SOL r	1.14 ± 0.14	1.11 ± 0.13	1.14 ± 0.15	1	1.12 ± 0.16	1.13 ± 0.16	1.13 ± 0.15	3%	0.28	0.04
PRE GM r	1.12 ± 0.11	1.10 ± 0.11	1.12 ± 0.13	1	1.10 ± 0.12	1.13 ± 0.17	1.11 ± 0.14	6%	0.17	0.02
PRE TA r	1.12 ± 0.10	1.09 ± 0.10	1.11 ± 0.12	1	1.09 ± 0.12	1.10 ± 0.12	1.11 ± 0.12	2%	0.46	0.02
SLR SOL l	**1.03 ± 0.15**	**1.07 ± 0.18**	**1.00 ± 0.16**	**1**	**0.95 ± 0.15**	**0.97 ± 0.08**	**0.92 ± 0.28**	**5%**	**0.04**	**0.09**
SLR GM l	1.04 ± 0.11	1.02 ± 0.10	1.02 ± 0.12	1	1.00 ± 0.14	0.96 ± 0.13	0.93 ± 0.18	3%	0.06	0.05
SLR TA l	**1.01 ± 0.20**	**0.97 ± 0.16**	**0.95 ± 0.19**	**1**	**1.13 ± 0.16**	**1.25 ± 0.19**	**1.34 ± 0.23**	**3%**	**<0.01**	**0.56**
SLR VM l	1.09 ± 0.08	1.07 ± 0.08	1.10 ± 0.10	1	1.10 ± 0.14	1.07 ± 0.10	1.08 ± 0.10	4%	0.35	0.03
SLR BF l	1.09 ± 0.14	1.05 ± 0.15	1.06 ± 0.15	1	1.09 ± 0.14	1.10 ± 0.15	1.10 ± 0.13	3%	0.47	0.02
SLR SOL r	**1.11 ± 0.12**	**1.08 ± 0.13**	**1.10 ± 0.15**	**1**	**1.18 ± 0.09**	**1.20 ± 0.14**	**1.30 ± 0.13**	**8%**	**0.02**	**0.19**
SLR GM r	**1.12 ± 0.11**	**1.10 ± 0.11**	**1.13 ± 0.12**	**1**	**1.09 ± 0.10**	**1.26 ± 0.14**	**1.33 ± 0.13**	**4%**	**0.04**	**0.14**
SLR TA r	0.99 ± 0.09	1.02 ± 0.09	0.97 ± 0.12	1	0.95 ± 0.17	0.99 ± 0.09	0.97 ± 0.10	2%	0.74	0.02
MLR SOL l	1.02 ± 0.15	1.05 ± 0.18	1.00 ± 0.17	1	0.93 ± 0.17	0.96 ± 0.26	0.99 ± 0.27	4%	0.88	0.01
MLR GM l	0.95 ± 0.13	0.97 ± 0.11	1.02 ± 0.14	1	0.96 ± 0.17	1.04 ± 0.30	1.02 ± 0.15	4%	0.91	0.01
MLR TA l	**0.84 ± 0.18**	**0.90 ± 0.13**	**0.94 ± 0.20**	**1**	**1.14 ± 0.18**	**1.21 ± 0.38**	**1.33 ± 0.18**	**5%**	**<0.01**	**0.42**
MLR VM l	1.10 ± 0.10	1.19 ± 0.18	1.09 ± 0.10	1	1.05 ± 0.09	1.13 ± 0.28	1.07 ± 0.09	4%	0.16	0.03
MLR BF l	1.11 ± 0.15	1.24 ± 0.11	1.15 ± 0.13	1	1.12 ± 0.13	1.15 ± 0.32	1.10 ± 0.12	3%	0.27	0.04
MLR SOL r	**0.91 ± 0.15**	**0.90 ± 0.17**	**0.95 ± 0.17**	**1**	**1.23 ± 0.20**	**1.20 ± 0.30**	**1.27 ± 0.14**	**5%**	**<0.01**	**0.44**
MLR GM r	**0.94 ± 0.14**	**0.99 ± 0.12**	**1.02 ± 0.15**	**1**	**1.13 ± 0.11**	**1.21 ± 0.27**	**1.23 ± 0.14**	**3%**	**0.01**	**0.32**
MLR TA r	0.93 ± 0.14	1.01 ± 0.12	0.97 ± 0.14	1	0.94 ± 0.12	0.90 ± 0.31	0.82 ± 0.14	6%	0.08	0.18
LLR SOL l	**1.03 ± 0.16**	**1.09 ± 0.16**	**1.02 ± 0.18**	**1**	**0.90 ± 0.18**	**0.84 ± 0.10**	**0.81 ± 0.26**	**4%**	**0.02**	**0.24**
LLR GM l	**1.12 ± 0.11**	**1.10 ± 0.08**	**1.13 ± 0.14**	**1**	**0.97 ± 0.12**	**0.96 ± 0.17**	**0.90 ± 0.14**	**3%**	**0.04**	**0.16**
LLR TA l	**1.18 ± 0.17**	**1.07 ± 0.08**	**1.05 ± 0.09**	**1**	**1.15 ± 0.20**	**1.25 ± 0.20**	**1.35 ± 0.22**	**5%**	**<0.01**	**0.47**
LLR VM l	1.09 ± 0.08	1.00 ± 0.09	1.10 ± 0.10	1	1.08 ± 0.12	1.07 ± 0.10	1.08 ± 0.09	5%	0.53	0.02
LLR BF l	**1.12 ± 0.13**	**1.03 ± 0.08**	**1.14 ± 0.15**	**1**	**1.04 ± 0.15**	**1.08 ± 0.10**	**1.21 ± 0.17**	**3%**	**0.04**	**0.13**
LLR SOL r	**1.10 ± 0.10**	**1.00 ± 0.10**	**1.13 ± 0.14**	**1**	**1.21 ± 0.14**	**1.44 ± 0.19**	**1.52 ± 0.22**	**6%**	**<0.01**	**0.48**
LLR GM r	**0.98 ± 0.13**	**1.00 ± 0.16**	**1.03 ± 0.09**	**1**	**1.17 ± 0.10**	**1.20 ± 0.15**	**1.37 ± 0.23**	**2%**	**<0.01**	**0.32**
LLR TA r	0.96 ± 0.14	1.00 ± 0.08	1.02 ± 0.08	1	1.03 ± 0.11	0.98 ± 0.11	0.94 ± 0.13	4%	0.30	0.03


*EMG amplitudes of all subjects (mean ± SD, *n* = 6 subjects) for the five recorded muscles of the left leg and three recorded muscles of the right leg during four phases (PRE 100 ms before treadmill perturbation until perturbation onset, SLR 30–60 ms, MLR 60–85 ms, LLR 85–120 ms after perturbation). Values are normalized to the iEMG during MVC. The values for hypo- and hyper-gravity are expressed in percent of the 1 g condition. For every leg, muscle and phase the Friedman test was applied to test the effect of the g-level on the EMG amplitude. The *p*-value refers to the Friedman Test (significant g-effects were labeled with bold letters) and $\eta_{p}^{2}$ to the effect sizes. Standard errors (SEs) over all seven gravity levels are illustrated.*

**FIGURE 5:**
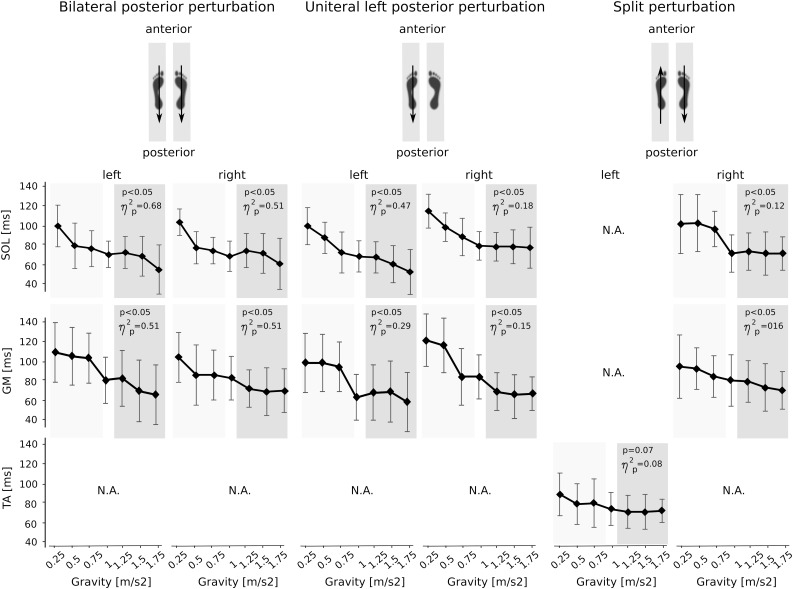
Muscle activation onset latencies for the plantarflexors [M. soleus (SOL) and M. gastrocnemius medialis (GM)] and the dorsiflexor [M. tibialis anterior (TA)] displayed for the seven gravity levels that span from hypo- to Earth to hyper-gravity. Values are displayed in ms. Independent of the postural set (bi- unilateral left or split perturbation), onset latencies shortened progressively with increasing gravity. Note that even the contralateral non-displaced leg (right leg during unilateral left perturbations) showed EMG bursts in response to the left leg’s perturbation, however, with longer onset latencies compared to the displaced leg. N.A. denotes the muscles that were not affected by the perturbation and thus did not show onset latencies. The *p*-value refers to the Friedman Test; $\eta_{p}^{2}$ displays the effect sizes.

After perturbation, compensatory neuromuscular responses in the leg musculature to external stimuli were gravity-sensitive. With progressively increased gravitation the neuromuscular activity increased. This was true for all three modes of perturbation (Table 1) and leg segments, and independent of the muscle’s function distinguished by flexors or extensors (Tables 2–4). As such, the neuromuscular activation intensity increased significantly and gradually as a function of gravity (Figure 1, 5). This increase was phase-specific, more pronounced for MLR and LLR (Figure 3) and less visible in the SLR. The M. soleus and M. gastrocnemius medialis were most affected by changes in gravity. In a few muscles, the rise in EMG amplitudes reached saturation and showed an asymptotic behavior or even a minor decline in the highest gravity level (>1.5 g). During unilateral left surface perturbations, the contralateral musculature of the non-displaced leg demonstrated a significant increase in neuromuscular activity with increasing gravity indicating an interlimb-synchronization for the MLR and LLR.

### Muscle Onset Latencies

Gravity-induced changes in the muscle onset latencies are illustrated in Figure 5. Activation onsets occurred only in the muscles that counteracted the perturbation stimulus. With increasing gravity, onset latencies were significantly reduced in the affected muscles involving the SOL, GM, and TA for the perturbed and not perturbed leg. The gravity-associated reduction in onset latency was also visible in the contralateral non-displaced leg, however, the neuromuscular activation in the contralateral leg was delayed as compared to the perturbed leg (*p* < 0.05).

### Antagonistic Co-activation Prior to Perturbation Onset

**FIGURE 6:**
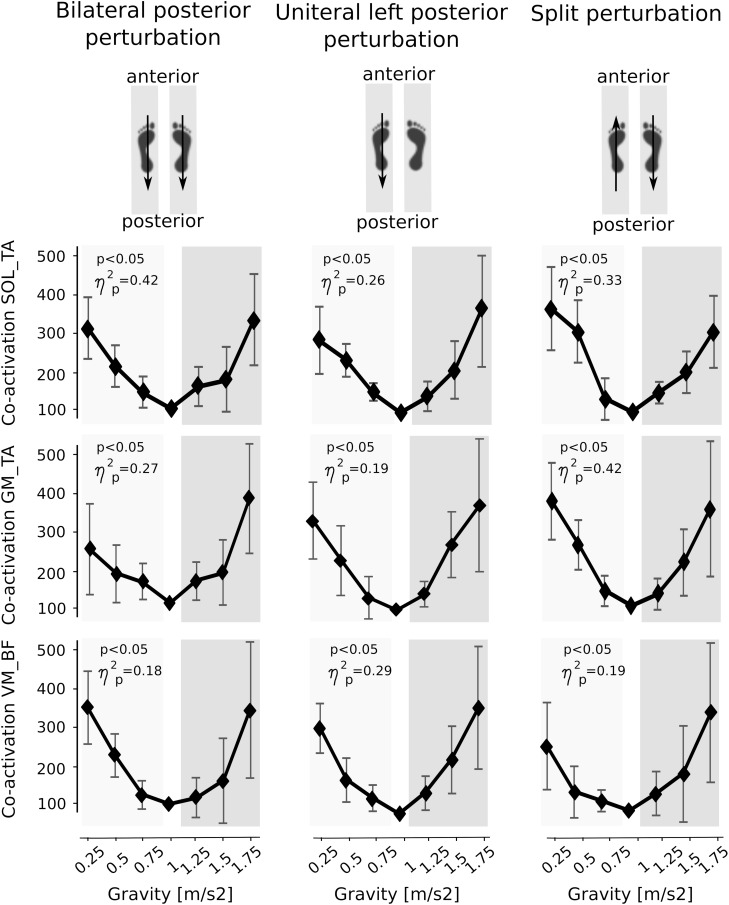
Muscle co-activation of antagonists encompassing the ankle (top and middle) and knee joint (bottom). Graphs illustrate the increase in co-activation above and below Earth gravity. Data are presented illustrate the co-activation of the M. soleus and M. tibialis anterior (SOL_TA), the M. gastrocnemius medialis and M. tibialis anterior (GM_TA) and the M. vastus medialis and M. biceps femoris muscles (VM_BF) for the left leg during the pre-activation phase prior to perturbation. The *p*-value refers to the Friedman Test; $\eta_{p}^{2}$ displays the effect sizes.

Means of the co-activation in PRE of antagonistic muscles encompassing the ankle and knee joint are illustrated in Figure 6. With both increasing and decreasing gravity above and below 1 g, the antagonistic co-activation increased significantly in the shank and thighs and equally for leg extensors and flexors prior to perturbation compared to the reference values of 1 g indicating a joint stiffening.

### Kinematics

**FIGURE 7:**
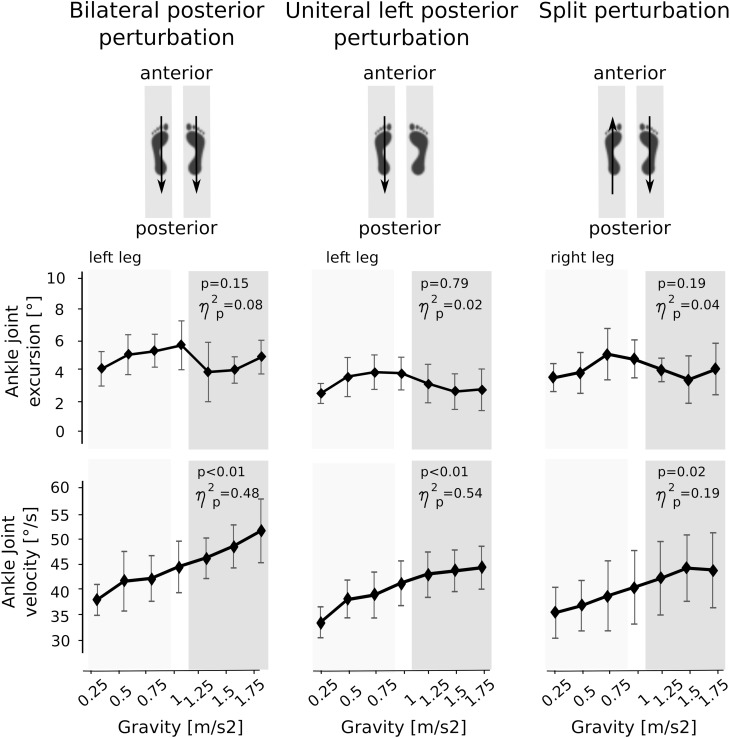
Means of the kinematic parameters for the seven gravity levels that span from hypo- to Earth to hyper-gravity. Adaptation in peak angular excursion of the ankle joint are displayed on the top, while changes in mean angular velocities of the ankle joint are illustrated on the bottom. Whereas angular excursions showed no significant changes over time, the angular velocities progressively increased with increasing gravity. The *p*-value refers to the Friedman Test; $\eta_{p}^{2}$ displays the effect sizes.

Means of the ankle and knee joint kinematics are displayed in Table 5. Mean signal traces of the ankle and knee joint excursions of a representative subject collected from a minimum of five perturbations for each gravity level are provided in Figure 1. Ankle and knee joint position at perturbation onset and maximal joint defections revealed no statistical differences between the gravity levels. The v_joint_ of ankle joint deflection increased progressively with increasing gravity (Figure 7).

**Table 5:** Kinematics for seven gradually and equidistantly increasing g-levels.

	Hypo gravity			Earth gravity	Hyper gravity			Statistics		
				
Bilateral posterior perturbation	0.25 g	0.5 g	0.75 g	1 g	1.25 g	1.5 g	1.75 g	*SE*	*P*	$\eta_{p}^{2}$
L ankle angle at perturbation onset (°)	94 ± 2	93 ± 2	93 ± 2	94 ± 2	94 ± 3	93 ± 3	95 ± 3	2%	0.91	0.01
R ankle angle at perturbation onset (°)	90 ± 4	91 ± 4	90 ± 3	92 ± 3	89 ± 4	92 ± 4	93 ± 5	1%	0.29	0.04
L max. ankle angular excursion (°)	-4 ± 1	-5 ± 1	-5 ± 1	-6 ± 2	-4 ± 2	-4 ± 1	-4 ± 2	2%	0.15	0.08
R max. ankle angular excursion (°)	-5 ± 1	-5 ± 1	-6 ± 1	-6 ± 2	-5 ± 1	-5 ± 1	-5 ± 1	1%	0.29	0.03
L knee angle at perturbation onset (°)	0 ± 5	0 ± 4	-1 ± 3	-2 ± 2	-1 ± 4	-1 ± 4	-1 ± 4	2%	0.80	0.01
R knee angle at perturbation onset (°)	2 ± 1	3 ± 2	1 ± 3	1 ± 3	1 ± 2	1 ± 1	1 ± 1	1%	0.76	0.02
L max. knee angular excursion (°)	2 ± 1	2 ± 1	2 ± 1	3 ± 2	2 ± 2	2 ± 1	2 ± 2	2%	0.15	0.07
R max. knee angular excursion (°)	2 ± 1	2 ± 1	2 ± 1	2 ± 2	2 ± 1	2 ± 1	2 ± 2	1%	0.29	0.03

**Unilateral left posterior perturbation**	**0.25 g**	**0.5 g**	**0.75 g**	**1 g**	**1.25 g**	**1.5 g**	**1.75 g**	***SE***	***P***	$\eta_{p}^{2}$

L ankle angle at perturbation onset (°)	94 ± 2	94 ± 2	93 ± 1	94 ± 2	93 ± 2	95 ± 3	94 ± 6	3%	0.65	0.03
R ankle angle at perturbation onset (°)	92 ± 5	91 ± 4	90 ± 3	92 ± 3	91 ± 2	91 ± 3	93 ± 4	2%	0.40	0.07
L max. ankle angular excursion (°)	-3 ± 1	-4 ± 1	-4 ± 1	-4 ± 1	-4 ± 1	-3 ± 1	-3 ± 1	3%	0.79	0.02
R max. ankle angular excursion (°)	-0 ± 0	-1 ± 1	-0 ± 0	-1 ± 1	-1 ± 1	-1 ± 1	-1 ± 1	1%	0.44	0.02
L knee angle at perturbation onset (°)	2 ± 4	0 ± 4	1 ± 3	1 ± 2	1 ± 2	1 ± 3	1 ± 2	2%	0.52	0.03
R knee angle at perturbation onset (°)	2 ± 1	2 ± 2	1 ± 3	1 ± 2	2 ± 3	1 ± 2	1 ± 2	2%	0.19	0.04
L max. knee angular excursion (°)	2 ± 1	3 ± 1	3 ± 1	3 ± 2	3 ± 2	2 ± 1	2 ± 1	3%	0.73	0.03
R max. knee angular excursion (°)	0 ± 1	1 ± 1	1 ± 1	1 ± 1	1 ± 1	0 ± 0	1 ± 1	1%	0.48	0.05

**Split perturbation (left anterior right posterior)**	**0.25 g**	**0.5 g**	**0.75 g**	**1 g**	**1.25 g**	**1.5 g**	**1.75 g**	***SE***	***P***	$\eta_{p}^{2}$

L ankle angle at perturbation onset (°)	92 ± 6	90 ± 2	90 ± 2	90 ± 1	90 ± 2	91 ± 3	93 ± 2	4%	0.16	0.07
R ankle angle at perturbation onset (°)	91 ± 4	91 ± 4	90 ± 3	92 ± 3	89 ± 4	93 ± 4	93 ± 4	1%	0.59	0.02
L max. ankle angular excursion (°)	6 ± 1	6 ± 1	7 ± 1	7 ± 1	5 ± 1	5 ± 1	6 ± 2	2%	0.49	0.02
R max. ankle angular excursion (°)	-3 ± 1	-4 ± 1	-5 ± 2	-5 ± 2	-4 ± 1	-4 ± 1	-4 ± 2	3%	0.19	0.04
L knee angle at perturbation onset (°)	2 ± 3	0 ± 4	-1 ± 3	0 ± 4	1 ± 5	1 ± 4	0 ± 6	4%	0.47	0.02
R knee angle at perturbation onset (°)	2 ± 1	3 ± 3	1 ± 4	0 ± 3	0 ± 3	1 ± 2	1 ± 2	4%	0.15	0.04
L max. knee angular excursion (°)	2 ± 1	3 ± 1	3 ± 1	2 ± 1	2 ± 1	2 ± 0	3 ± 2	4%	0.82	0.01
R max. knee angular excursion (°)	1 ± 1	2 ± 1	2 ± 2	2 ± 3	1 ± 1	1 ± 1	2 ± 1	2%	0.47	0.02


*Mean ankle and knee joint angles at perturbation onset and maximal ankle and knee angular excursions of the left (l) and right (r) leg after perturbation. Postural set was manipulated by either bilateral posterior (top), unilateral left posterior (middle) and split perturbation (bottom), each mean value comprises *n* = 6 participants. For every joint angle of each leg the Friedman test was applied to test the effect of the g-level on the kinematic parameters. The *p*-value refers to the Friedman Test and $\eta_{p}^{2}$ to the effect sizes. Standard errors (SEs) over all seven gravity levels are illustrated.*

### Correlations Between Joint Velocities and Neuromuscular Activity

**FIGURE 8:**
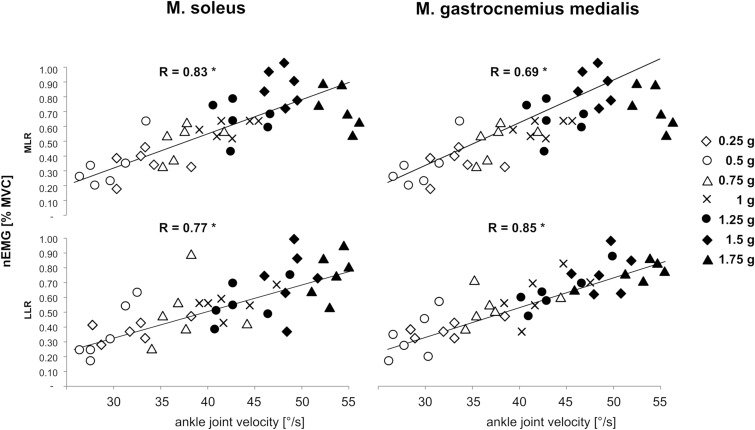
Gravity-dependent bivariate correlations and correlation coefficients among the variable angular velocity of the ankle joint (abscissa) with the normalized EMGs of M. soleus (left leg) and M. gastrocnemius medialis (right leg) in the relevant EMG phases Medium-latency response (MLR, 60–85 ms after perturbation onset) and long-latency response (LLR, 85–120 ms after perturbation onset, ordinate) during left posterior perturbation. Findings revealed that the EMG was positively correlated to ankle joint velocity (^∗^ indicate significant findings *p* < 0.05).

A significant positive correlation was detected for the variable v_joint_ of the ankle with muscle activity in the SOL and GM in the reflex phases MLR and LLR for bi-lateral posterior perturbations. Corresponding graphs to illustrate bivariate correlations and correlation coefficients for bilateral perturbations are displayed in Figure 8.

## Discussion

With the success of re-gaining postural equilibrium after its perturbation, this study provides a major insight into the gravity-dependency of postural recovery responses after translational surface perturbations. Findings reveal increased muscle activity and co-contraction in the lower extremities prior to perturbation in hypo- and hyper-gravity. After perturbation, gradually reduced muscle activation onset latencies and increased neuromuscular activation in the MLR and LLR were manifested with a progressive rise in gravity. Neuro-mechanical adaptations to gravity were more distinct and muscle onset latencies were shorter in the displaced compared to the non-displaced leg. Ankle and knee joint deflections remained unaffected, whereas angular velocities increased with increasing gravitation. Positive correlations were manifested for angular velocities and EMG amplitudes of SOL and GM for the MLR and LLR. Effects were more pronounced in bi- compared to unilateral or split perturbations.

**FIGURE 9:**
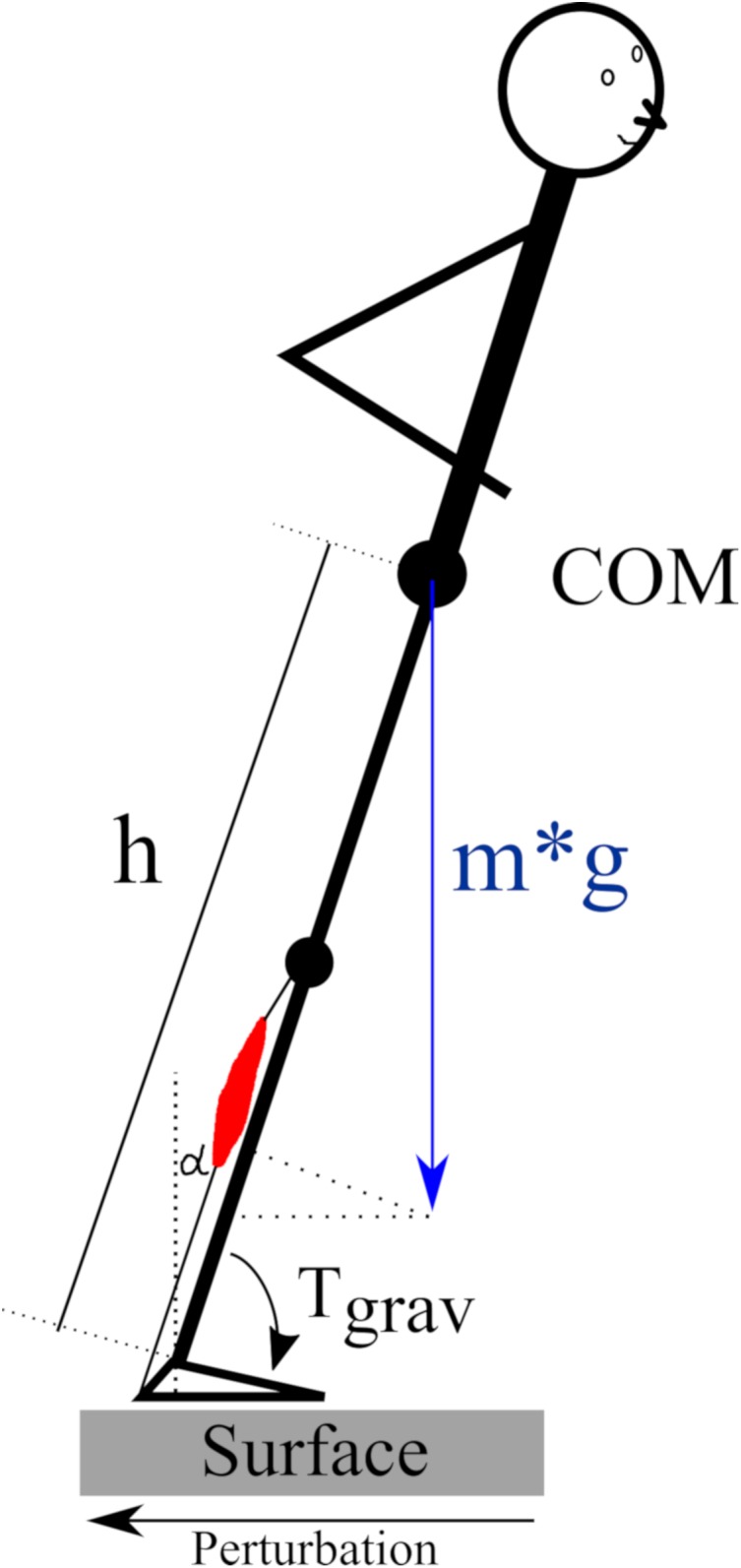
Schematic of the gravity-dependency of the human body in the sagittal plane according to the inverted pendulum model published by van der Kooij et al. (2005) and Bottaro et al. (2008). The pendulum is inclined in the anterior direction due to a translational posterior surface perturbation. The required muscle force of the plantarflexors (red) for a repositioning of the center of mass (COM) above the base of support depends on the body mass (*m*), the COM height (*h*) above the ankle joint and the inclination angle alpha (α) with reference to the vertical. The gravity-dependent ankle joint torque *T*_grav_ = *m*^∗^*g*^∗^*h*^∗^sin(α).

The regulation of human posture in our terrestrial habitat is based on a physiological model (Taube et al., 2008) that involves an accurate coordination of muscle onset and activation patterns between the two legs and their segments (Clement et al., 1984; Dietz et al., 1989a; Rosa, 2015). Slips or stumbles in particular require precise neuronal control of skeletal muscles transmitting the force to the skeleton in order to regain postural equilibrium after its deterioration. At this point, it is important to consider the role of gravity: Counteracting the gravitational force in the vertical plane and compensating for an immediate deterioration of posture control caused by COM shifts in the horizontal plane presupposes an adequate level of activation of the muscle, which may depend on the loading force (Figure 9) (Nakazawa et al., 2004; Ritzmann et al., 2015). With reference to gravitational variation, the underlying neuromechanical coupling (Mergner and Rosemeier, 1998) and its proportionality to the ankle joint torque, (Gollhofer et al., 1989) we wish to highlight three major aspects of these concepts:

### Timing and Magnitude of Neuromuscular Responses as an Adaptation to Gravity

Our findings indicate that postural perturbations can be counteracted intuitively, appropriate muscle activity can be anticipated, and segmental and COM positioning can be properly adjusted. Subjects adapted their motor control pattern progressively, even though planetary acceleration profiles differ largely between Earth, hyper- and hypo-gravity. With increasing gravity, the EMG amplitudes increased and muscle activation onset latencies diminished. This was true for all muscles counteracting the perturbation, and valid for all types of perturbation modes (Table 2) and leg segments, and were independent of muscle function distinguished by flexors or extensors (Tables 2–4). The most prominent gravity-induced adaptations were observed in the MLR, which is considered to be governed by supraspinal structures via the brain (Taube et al., 2006) (Figure 3). It is assumed that, beyond the massive increase in load, which in turn immediately increases the torques affecting the body, the altered vestibular input results in an excitatory influence exerted by the vestibular organ on muscle and tendon receptors (Lackner and DiZio, 2000). In this context, an increased vestibulo-spinal influence on the excitation of alpha and gamma motoneurons has been shown to be related with the function of the antigravity musculature (Lackner and DiZio, 1993; Kalb and Solomon, 2007). Thus, our physiological model moves functional reflexes into the focus and underlines that the gravity-adjustment under supraspinal control serves as a successful management of posture control preventing falling.

In a few distal muscles, the rise of the neuromuscular activity for MLR and LLR reached saturation and showed an asymptotic behavior or even a minor decline in the highest gravity level (>1.5 g) (Figure 3). We expect that this phenomenon could be attributed to neuronal inhibition initiated by supraspinal centers of the CNS (Dietz, 1999; Winters and Crago, 2012; Aagaard, 2018). Extreme gravity levels exceeding 2 g are difficult to tolerate and compensatory motor control in fall-simulations is even more critical in these conditions (Figure 9). We can only speculate in terms of the underlying mechanisms that may be found in a segmental shift from distal to proximal body segments, as has been previously reported (Ritzmann et al., 2015).

### Neuro-Mechanical Considerations

The positive correlations between muscle activation intensities and the v_joint_ in the ankle emphasizes the neuromechanical coupling which may be determined by gravity (Dietz et al., 1989b; Duysens et al., 2000). As indicated by simulations that utilize the inverted pendulum model to describe the habitual orthograde human posture, ankle joint torque caused by perturbations increased proportional to the gravitational loading (Figure 9) (van der Kooij et al., 2005; Bottaro et al., 2008). With reference to the study of Dietz et al. (1989a), it is expected that the neuromuscular responses must increase proportionally to efficiently counteract the perturbation with adequate muscle force. These considerations underline the findings of the current study that highlight an interrelationship between joint mechanics and neuromuscular attributes, and could also be confirmed in experiments using water buoyancy (Dietz et al., 1989b; Minetti et al., 2012b). Within this physiological model, authors postulated the existence of a load receptor system detecting changes in gravity, allowing the integration of this sensory information within the CNS leading to an adequate adjustment of muscle responses and associated joint torques to re-gain a stable posture after deterioration (Dietz, 1998; Dietz and Duysens, 2000; Bachmann et al., 2008).

### Inter-Limb Coordination

Biomechanical models highlight the importance of harmonized inter-limb coordination during perturbed or unperturbed stance (Dietz et al., 1989a) and gait in healthy populations (Russell et al., 2010; Fujiki et al., 2015). It is well in line with previous publications (Dietz et al., 1989a) that leg symmetries were not perfectly synchronous, but showed a small delay in onset latency of approximately 15 ms and slightly diminished amplitudes in the shank musculature (Figure 5). The temporal and directional synchronization of activation intensities in both limbs was shown to be paramount in the regulation of the COM within its base of support to safely maintain upright posture in bipedal stance or locomotion (Dietz et al., 1989a; Dietz, 1996; Habib Perez et al., 2016). Changes in gravity may have made the matching of contralateral movement even more important, particularly in conditions in which the subjects were exposed to high gravitation loads (>1 g). For example, unilateral left perturbations directed backward were followed by a bilateral gastrocnemius-EMG response in the left and right leg, and a forward-directed perturbation by a bilateral tibialis anterior-EMG response. However, the first EMG rise in the contralateral non-displaced leg occurred later and was smaller, thus, it may have contributed less to regain a stable posture after deterioration (Dietz et al., 1989a).

### Limitation

For a conclusive statement, it is crucial to consider the limitations of the study. Three aspects are of substantial importance; the first one deals with stimulus prediction, the second one with the experimental setting in the parabolic flight and the third one with the generalization of our findings. First, although the perturbation direction, bilateralism and the duration of the pauses have been randomized among the trials (Dietz et al., 1989a), the subjects were aware that a perturbation would come. Despite the unknowledge about the perturbation characteristics, we cannot fully exclude that the subjects pre-activated or co-contracted the muscles in an effort to be prepared for any disturbance of posture control. Second, data collection has been executed during parabolic flight maneuvers. Although the order of the gravity levels has been randomized and the selection criteria for valid attempts and the gravity span have been rigorously pre-defined; parabolic flights are test flights and the time intervals for the data collection are short. They could also include small changes in gravity, which are not existent on the International Space Station. In addition, only a limited number of trials could be collected due to the restricted number of parabolas (Pletser et al., 2012). Third, although our findings highlight adaptability to various gravitation conditions, they cannot be generalized to all types of imposed motion. Besides the wearing of space suits limiting angular excursion in the limb joints (Schmidt, 2001; Gernhardt et al., 2008) and of vision-restricting helmets (Schmidt, 2001), lunar or Martian dust (Gaier, 2018), low friction coefficients (Sperling, 1970; Minetti et al., 2012b) or other surface particularities (Pavei and Minetti, 2016) are likely to impede habitual orthograde stance control on other planets as well.

## Conclusion

In view of upcoming space missions to the Moon and Mars (Minetti, 2001), the control of posture and locomotion under variable gravitation is paramount. The findings of the current study give a unique insight into neuromuscular regulation of human orthograde stance as a function of gravity. The CNS demonstrated remarkable adaptability to compensate for the sudden deterioration of postural balance among gravity levels spontaneously switching between 0 g and 2 g. This includes the systematic up- and down-regulation of muscle activity and muscle activation onset latencies accompanied by synchronized inter-limb coordination with the success of regaining postural equilibrium in each of the recorded gravity levels.

These results are of functional relevance in view of foreseen interplanetary manned space explorations: first, by integrating habituation sessions in the Astronauts 5-years preparation process and second, by establishing new therapy and space-relevant training modalities addressing particular strategies and adaptations by means of over- or under-loading conditions (Freyler et al., 2014).

## Ethics Statement

All participants gave written informed consent to the experimental procedure, which was in accordance with the latest revision of the Declaration of Helsinki and approved by the French authorities responsible for the protection of subjects participating in biomedical research (DEMEB of the AFSSAPS) as well as the ethics committee of the University of Freiburg (89/12). The participants underwent two obligatory medical investigations and were healthy with no previous neurological irregularities or injuries of the lower extremities. The experiments comply with current German laws and were approved by the local ethics committee.

## Author Contributions

RR, KF, and AG: conceptualization and funding acquisition. RR, KF, MH, JH, and JK: investigation, methodology, resources, software, and review and editing. RR and KF: project administration. RR and AG: supervision. RR: writing of the original draft and contributed to figures and tables.

## Conflict of Interest Statement

The authors declare that the research was conducted in the absence of any commercial or financial relationships that could be construed as a potential conflict of interest.
